# Targeting CD276 for T cell-based immunotherapy of breast cancer

**DOI:** 10.1186/s12967-024-05689-4

**Published:** 2024-10-04

**Authors:** Ilona Hagelstein, Laura Wessling, Alexander Rochwarger, Latifa Zekri, Boris Klimovich, Christian M. Tegeler, Gundram Jung, Christian M. Schürch, Helmut R. Salih, Martina S. Lutz

**Affiliations:** 1 Clinical Collaboration Unit Translational Immunology, Department of Internal Medicine, German Cancer Consortium (DKTK), University Hospital Tübingen, Otfried-Müller-Str. 10, 72076 Tübingen, Germany; 2 Cluster of Excellence iFIT (EXC 2180) “Image-Guided and Functionally Instructed Tumor Therapies”, University of Tübingen, Tübingen, Germany; 3 Department of Pathology and Neuropathology, University Hospital and Comprehensive Cancer Center Tübingen, Tübingen, Germany; 4 Department for Immunology and German Cancer Consortium (DKTK), Eberhard Karls University, Tübingen, Germany; 5 Department of Obstetrics and Gynecology, University Hospital Tübingen, Tübingen, Germany; 6 Department of Peptide-Based Immunotherapy, Institute of Immunology, University and University Hospital Tübingen, Tübingen, Germany

**Keywords:** Breast cancer, CD276, B7-H3, CD3, Bispecific antibody, T cell, Immunotherapy

## Abstract

**Background:**

Breast cancer (BC) is the most common malignancy in women. Immunotherapy has revolutionized treatment options in many malignancies, and the introduction of immune checkpoint inhibition yielded beneficial results also in BC. However, many BC patients are ineligible for this T cell-based therapy, others do not respond or only briefly. Thus, there remains a high medical need for new therapies, particularly for triple-negative BC. CD276 (B7-H3) is overexpressed in several tumors on both tumor cells and tumor vessels, constituting a promising target for immunotherapy.

**Methods:**

We analyzed tumor samples of 25 patients using immunohistochemistry to assess CD276 levels. The potential of CC-3, a novel bispecific CD276xCD3 antibody, for BC treatment was evaluated using various functional in vitro assays.

**Results:**

Pronounced expression of CD276 was observed in all analyzed tumor samples including triple negative BC. In analyses with BC cells, CC-3 induced profound T cell activation, proliferation, and T cell memory subset formation. Moreover, treatment with CC-3 induced cytokine secretion and potent tumor cell lysis.

**Conclusion:**

Our findings characterize CD276 as promising target and preclinically document the therapeutic potential of CC-3 for BC treatment, providing a strong rationale for evaluation of CC-3 in BC patients in a clinical trial for which the recruitment has recently started.

**Supplementary Information:**

The online version contains supplementary material available at 10.1186/s12967-024-05689-4.

## Background

Breast cancer (BC) is the most common cancer among women worldwide and is the second leading cause of female cancer mortality [1]. Despite substantial advances over the recent years, the still high rates of recurrence and mortality require further improvements of treatment options, as still up to 30% of women experience recurrence in advanced stages or metastatic disease [2, 3].

BC is classified into various molecular subtypes based on genetic characteristics and immunophenotypic features such as protein expression, primarily based on biological and clinicopathological features as luminal A, luminal B, HER2-enriched and basal-like or triple-negative BC (TNBC) [4]. The HER2 membrane receptor (CD340), which is encoded by the *ERBB2* proto-oncogene, is expressed in HER2-positive (HER2-enriched, HER2^+^) and a subset of luminal B BC [5]. Subtypes of HER2-negative (HER2^−^) BC, including luminal A, partial luminal B, and basal-like subtypes, demonstrate varying expression of hormone receptors (HR) for estrogen and progesterone [6]. HR-positive/HER2-negative (HR^+^/HER2^−^) BC is the most common subtype, representing two-thirds of all BC cases [7].

Treatment options for BC include surgical resection, radiotherapy, and systemic interventions such as chemotherapy and hormone therapy for estrogen and progesterone receptor-positive subtypes [8]. When it comes to immunotherapy, monoclonal antibodies (mAbs) such as trastuzumab and pertuzumab have significantly improved survival rates for HER2^+^ BC [9]. Another huge change in treatment was caused by the introduction of immune checkpoint inhibitor (ICI)-based immunotherapy into routine care [10, 11]. ICIs, for example pembrolizumab, achieved promising outcomes in the treatment of TNBC, highlighting the potential of T cell-based therapies in BC treatment [11, 12]. However, it is important to note that only about 5–20% of patients respond to this treatment [13], and treatment options particularly for TNBC are otherwise rather limited.

The transmembrane protein CD276 (B7-H3), a member of the immunomodulatory B7 family, recently receives great interest as target for cancer immunotherapy [14, 15]. CD276 is overexpressed over a great variety of solid tumors, has limited expression in healthy tissues [16], and is not only expressed on tumor cells themselves but also on tumor vessels. [17]. Expression has been linked to tumor cell migration, proliferation, invasion, and angiogenesis and has been implicated to influence tumor immune evasion, but its precise immunomodulatory function is not yet clear [18]. First reports have provided evidence that CD276 expression in BC, including TNBC, is associated with poor prognosis [19–22].

Bispecific antibodies (bsAbs) that stimulate CD3 on T cells and bind a tumor-associated antigen are increasingly entering cancer treatment. Notable examples of impressive clinical results with bsAbs include the CD20 and CD3 targeting bsAbs mosunetuzumab as well as glofitamab for the treatment of relapsed or refractory lymphoma [23–25] and the BCMA-directed constructs teclistamab as well as elranatamab for treatment of multiple myeloma [26, 27].

In solid tumors, T cell function frequently appears to be impeded, among others by insufficient T cell infiltration into the tumor site [28]. Targeting CD276, which is expressed not only on tumors cells but also on tumor vessels, may serve to overcome this obstacle. Targeting tumor vessels could improve access of immune effector cells to the tumor site, potentially overcoming a major challenge for T cell-based cancer immunotherapy. Recently, we reported on a novel CD276xCD3 bsAb termed CC-3 that we evaluated for treatment of colorectal and gastrointestinal cancers [29, 30]. Based on these data, CC-3 is presently being evaluated in a clinical trial (NCT05999396) to evaluate safety and efficacy in colorectal cancer patients. Here we assessed the therapeutic potential of CC-3 as a treatment option for BC.

## Methods

### Relative gene expression of CD276 based on TCGA database analysis

Data on the relative expression of CD276 in tumor tissue for BC patients (1805 cases) and corresponding normal tissue (112 cases) were extracted from the Cancer Genome Atlas (TCGA) database (http://www.oncolnc.org/) using the GTEx project. Additionally, information on CD276 expression regarding tumor stage and HER2 expression was obtained from TCGA through the GTEx project. The Gene Expression Profiling Interactive Analysis (GEPIA) web server (http://gepia.cancer-pku.cn/) was used to display the results, consistent as previously described [31].

### CD276 expression in breast cancer tissue

Freshly sectioned BC tissues (n = 25 cases) were formalin-fixed and paraffin-embedded (FFPE). Tumor samples were obtained from the biobank of the Institute of General and Molecular Pathology and Pathological Anatomy Tübingen. Immunohistochemical staining of the sections was performed on an automated VENTANA BenchMark ULTRA (Roche, Basel, Switzerland) according to the manufacturer's protocol, employing a routinely used HER2 antibody (clone 4B5, Roche) and the CD276 antibody RBT-B7H3 (Medac/Bio SB). BC sections were stained with hematoxylin and eosin (H&E). Sections were analyzed for HER2 and CD276 expression under the supervision of a board-certified pathologist (C.M.S.). The routinely used IHC-Score for HER2 staining was employed: 0 = negative; 1 +  = negative; 2 +  = weakly positive (equivocal); 3 +  = strongly positive [32]. Staining intensity of CD276 was evaluated using a standard scoring system: 0 = no expression; 1 = weak but detectable expression; 2 = moderate but clearly positive expression; 3 = strong expression [16]. In addition, the percentage of positively stained cells was assessed in two independent evaluations. H-scores were calculated by multiplying the percentage of positively stained cells by the staining intensity observed for each score.

### Cell lines and peripheral blood mononuclear cells (PBMC)

BC cell lines MCF-7 (luminal A-like), BT-474 (luminal B-like), SK-Br-3 (HER2^+^) and MDA-MB-468 (triple-negative) as well as HEK293T cells were from ATCC (American Type Culture Collection). The authenticity of the cells was regularly checked by validation of the respective immunophenotype as described by the supplier by means of flow cytometry. Contamination with mycoplasma of cell culture was routinely excluded at least every three months. Peripheral blood mononuclear cells (PBMC) of healthy donors were obtained after informed consent and isolated by density gradient centrifugation (Biocoll; Biochrom, Berlin, Germany), and monocytes within the PBMC were depleted using human CD14 MicoBeads UltraPure kit (Miltenyi Biotec, Bergisch-Gladbach, Germany). PBMC were viably frozen and stored in liquid nitrogen.

### Generation of CD276 knockout cells

CD276 was knocked out in MDA-MB-468 cells using the CRISPR-Cas9 system. The single guide RNA sequence (5′-CACAGGGCAACGCATCCCTG-3′) designed using the CRISPick online tool was cloned into the p-LCRISPR.EFS.PAC vector (Addgene plasmid # 57828). Subsequently, the lentiCRISPR p-LCRISPR.EFS.PAC-B7-H3 plasmid, along with pMD2.g (RBK243) (Addgene plasmid # 12259) and pSPAX2 (RBK242) (Addgene plasmid # 12260) plasmids, were co-transfected into HEK293T cells using TransIT (Mirus Bio, Madison, WY). The resulting lentivirus was used for transduction of MDA-MB-468 cells, which were then selectively cultured using 1 μg/ml puromycin (Sigma-Aldrich, St. Louis, MO).

### Production and purification of bispecific antibodies

The CD276xCD3 bsAb termed CC-3 and the respective isotype control (MOPCxCD3) without target specificity were previously described [33, 34]. Briefly, the constructs were generated in ExpiCHO cells (Gibco, Carlsbad, CA) and then purified from the culture supernatant by affinity chromatography using Mabselect affinity columns (GE Healthcare, Munich, Germany). Analytical and preparative size exclusion chromatography was performed using Superdex S200 Increase 10/300GL and HiLoad 16/60 columns (GE Healthcare). Using the EndoZyme II kit (BioMerieux, Marcy-l'Étoile, France) according to the manufacturer's instructions, it was ensured that endotoxin levels were < 0.5 EU/ml.

### Reporter cell assays

To determine the capacity of CC-3 to induce T cell responses, a T cell activation bioassay (Promega, Madison, WI) was used according to manufacturer’s instructions. Briefly, 50,000 BC cells per well were seeded the day before. Then CC-3 in a concentration range of 15–0.02 nM and TCR/CD3 Effector Cells (NFAT) were added followed by incubation for 6 h. To detect engagement of TCR/CD3, induced luminescence was measured with a Glomax Reader (Promega).

The Jurkat J76TPR reporter cell line was provided by Prof. Mirjam H.M. Heemskerk [35], and used to determine the induction of the transcription factors NFAT (GFP) and NF-κB (mCherry) in T cells upon binding of CC-3. For analysis of T cell activation, a coculture of tumor cells (100,000 cells/well) and Jurkat J76TPR cells (200,000 cells/well) was seeded with or without CC-3 in a concentration range of 10–0.0003 nM or with isotype control. After 24 h of incubation, expression of reporter genes was analyzed by flow cytometry.

The IncuCyte® S3 Live-Cell Analysis System (Essenbioscience, Sartorius, Göttingen) was used to perform a time kinetic analysis of the induction of reporter genes. BC cells were seeded in 96-well plates and cocultured with Jurkat J76TPR reporter cells (E:T ratio 2:1) with or without the indicated bsAbs (1 nM each). To determine the induction of reporter genes, images were taken every hour with a 20 × magnification. The transcription factors NFAT (GFP) and NF-κB (mCherry) positive cells were quantified by normalizing the counts of GFP or mCherry positive objects to the respective measurement at T = 0 h.

### Image stream

BC cells were incubated for 1.5 h with PBMC (E:T 2.5:1) with or without CC-3 or isotype control. After incubation, cells were stained with a FITC-labeled anti-human Fc-specific Ab (Jackson ImmunoResearch, West Grove, PA) to detect cell-bound CC-3 followed by surface staining with CD4-PECy7, CD8-BV605 and CD326-PE (all from BioLegend, San Diego, CA) and subsequently fixed in 1.5% paraformaldehyde (PFA) in PBS for 15 min at RT. Cells were then washed with PBS, followed by incubation with PBS-T (PBS + 0.1% Tween20) for 20 min at RT. Then, DAPI (0.1 µg/ml, Invitrogen, Carlsbad, CA) and Phalloidin-AF647 (1:500, Thermo Fisher Scientific, Waltham MA) to stain for nuclei and actin were added. At least 100.000 cells were collected with 40 × magnification using the amnis ImageStream mkII Imaging Flow cytometer (Cytek Biosciences, San Diego, CA). Data were analyzed with IDEAS® Image analysis software. All samples were gated on binuclear aggregates. Organized immunological synapses were identified by measuring the Co-localization between two images (single tumor cells and single T cells) in the overlapping region of polarized Phalloidin.

### Flow cytometry

For analysis of CD276 surface expression and titration of CC-3, cells were incubated with parental murine CD276 antibody (10 µg/ml) (clone 7C4) and CC-3 or the corresponding isotype controls followed by goat anti-mouse PE conjugate (Dako, Glostrup, Denmark) and goat anti-human PE conjugate (Jackson ImmunoResearch), respectively. To determine the number of CD276 molecules on the cell surface, quantitative immunofluorescence analysis was performed using the murine mAb 7C4 and the QIFIKIT kit (Dako) as previously described [36].

To evaluate T cell activation, degranulation and proliferation, staining with CD69-PE, CD107a-PE (BD Pharmingen) as well as CD4-APC, CD8-FITC, CD62L-PB, and CD45Ro-PeCy7 (BioLegend) was used.

7-AAD (BioLegend) was used for live- and dead-cell discrimination. Measurements were performed using a FACS Canto II or FACS Fortessa (BD Biosciences, San Diego, CA) and data was analyzed using FlowJo software (FlowJo LCC, Ashland, OR).

### T cell activation and degranulation assays

To determine activation (CD69) and degranulation (CD107a) of effector cells, tumor cells were cultured with PBMC (E:T 5:1) in the presence or absence of CC-3 or isotype control (1 nM). T cell activation was determined by flow cytometry after 24 h. To analyze T cell degranulation, cells were cultured for 4 h in the presence of CD107a-PE (1:25), BD GolgiStop and BD GolgiPlug (1:1000, both BD Biosciences) followed by flow cytometric analysis.

### T cell proliferation assays

PBMC (100,000/well) were seeded in triplicates in 96-well plates with increasing concentrations of CC-3 or corresponding control and with irradiated (30 Gy) BC cells (50,000/well). After incubation for 48 h, cells were pulsed with ^3^H-methyl-thymidine (0.5 μCi/well) for 20 h and harvested on filtermats. Incorporated radioactivity was determined by liquid scintillation counting in a 2450 Microplate counter (Perkin Elmer, Waltham, MA).

To analyze proliferation of T cell (memory) subsets, PBMC were incubated with tumor cells (E:T 5:1) in the presence or absence of CC-3 or isotype control (1 nM). Fresh target cells were added and the treatment was repeated after three days. T cell subsets were analyzed for expression of CD4, CD8, CD45ro and CD62L by flow cytometry after 6 days.

### Analysis of cytokine secretion

To determine bsAb-induced cytokine release, PBMC were cultured with BC cells (E:T ratio 5:1) in the presence or absence of CC-3 or control (1 nM each). After 24 h, supernatants were collected and analyzed for release of IL-2, IFN-γ and TNF by Legendplex assays (BioLegend) according to the manufacturer’s protocol.

### Cytotoxicity assays

To determine target cell lysis through flow cytometry-based assays, tumor cells were loaded with 2.5 µM CellTrace™ Violet (Thermo Fisher Scientific) and cultured with PBMC (E:T 5:1) in the presence or absence of CC-3 or isotype control (1 nM each). Standard calibration beads (Sigma-Aldrich) were used to assure consistent test volumes and to account for the number of living target cells.

For analysis of long-term cytotoxicity, BC cells were cultured with PBMC (E:T ratio 5:1) in the presence or absence of CC-3 bsAb or control (1 nM). The xCELLigence RTCA system (Roche Applied Science, Penzberg, Germany) was used to conduct real-time cytotoxicity analysis for a time period of 120 h.

### Statistics

If not otherwise indicated, values depict means ± standard deviation (SD). Student’s t test, Mann‐Whitney U test, one‐way ANOVA and Friedman’s test was used for continuous variables. If significant differences by ANOVA were found, group wise comparison was done (Tukey’s multiple comparison test). If significant differences were found by Friedman’s test, Dunn’s multiple comparisons test was used. All statistical tests were considered statistically significant when *p* was below 0.05. Statistical analysis was performed using GraphPadPrism (v.8.1.0).

## Results

### CD276 is expressed in breast cancer regardless of HER2 status

**Fig. 1:**
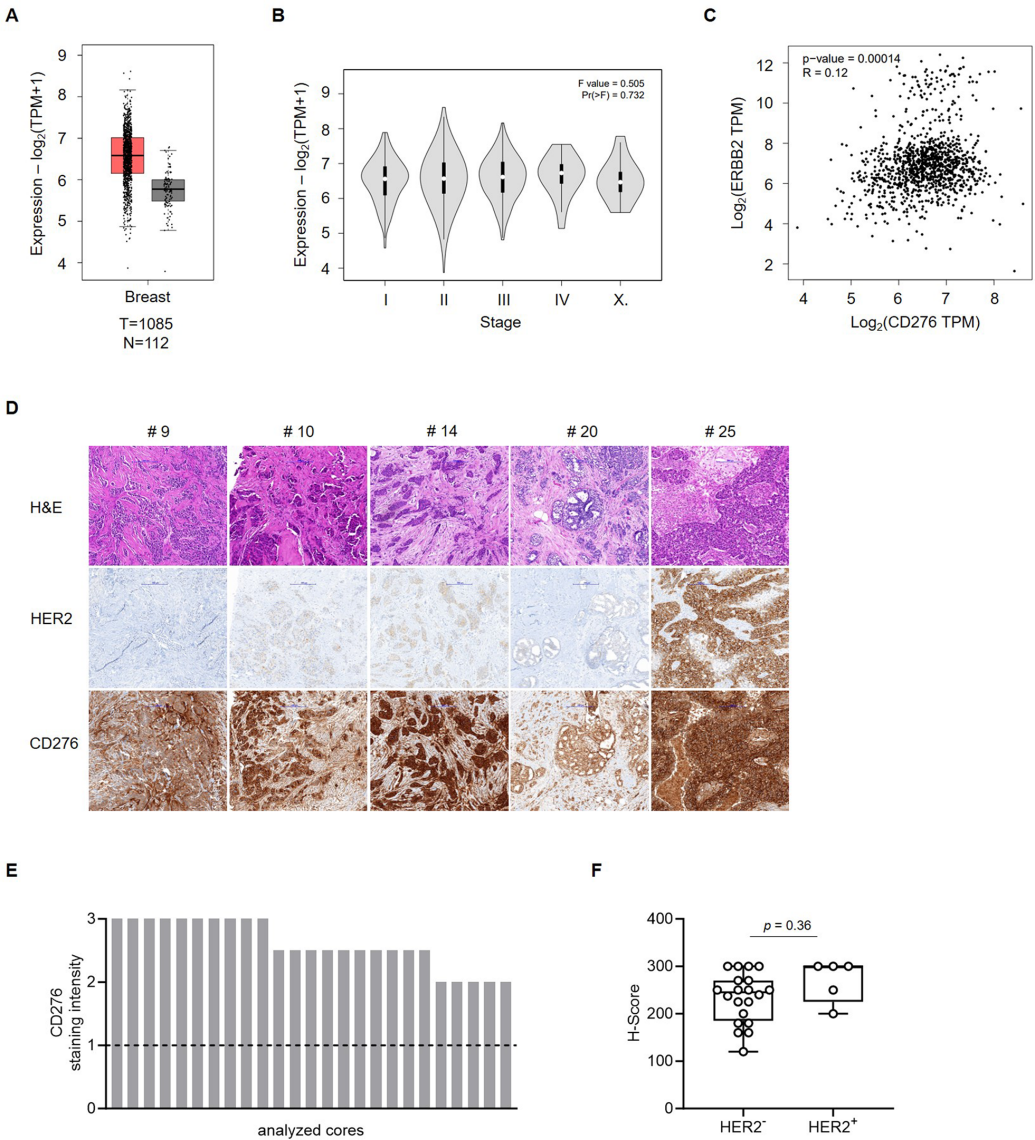
Expression of CD276 (B7-H3) in tumor tissue from BC patients. **A**–**C** The expression of CD276 mRNA in BC and normal breast tissues from TCGA datasets was evaluated by the online tool GEPIA. **A** Relative gene expression profile of CD276 in tumor (T) and normal (N) breast tissues. **B** Expression of CD276 in BC cases at various disease stages (I-IV, X for not specified).** C** Correlation of CD276 with HER2 gene expression in BC. **D**–**F** Freshly cut tissues of BC patients (n = 25 cases in total) were formalin fixed and paraffin embedded. Sections were analyzed for HER2 and CD276 expression by immunohistochemistry. Staining intensity was assessed under the supervision of a board-certified pathologist. IHC-Score for HER2 staining: 0 = negative; 1 +  = negative; 2 +  = weakly positive (equivocal); 3 +  = strongly positive. CD276 staining intensity: 0 = no expression; 1 = weak, but detectable expression; 2 = intermediate, but clearly positive expression; 3 = strong expression. **D** Representative images of tumor tissue sections after hematoxylin–eosin (H&E), HER2 and CD276 staining (10 × magnification). **E** CD276 expression in BC for n = 25 cases. Dotted line represents cut-off for positivity (CD276 staining intensity 1 = weak, but detectable expression). **F** H-Scores for CD276 depending on HER2 expression status were calculated by multiplying the % cells positive stained x the staining intensity seen for each score. Results are shown as mean ± SD

As a first step, we analyzed CD276 mRNA expression using TCGA datasets, which comprised 1085 samples of BC and 112 samples of healthy breast tissue. Our findings showed higher CD276 mRNA expression in BC tissues compared to healthy tissues (Fig. 1A). When comparing CD276 mRNA levels in the TCGA dataset stratified by BC disease stage (I–IV or X for not specified), no significant differences in expression levels were observed (Fig. 1B). Correlation analysis between HER2 and CD276 expression showed only a weak association between the two genes (Fig. 1C).

Next, tissue sections from 25 BC cases were immunohistochemically analyzed for CD276 expression. The included cases exhibited different TNM staging and some were treated with neoadjuvant therapy (Supplemental Table 1). All specimens showed membranous CD276 staining (Fig. 1D, Supplemental Figure S1 and Supplemental Table 2) with a minimum staining intensity of 2 + (moderate but clearly positive) with none of the cases being negative for CD276 (Fig. 1E). 40% (n = 10) of BC cases showed strong staining intensity (3 +), whereas the others (n = 15) were moderate but clearly positive (2 + and 2–3) (Table 1). There was no significant difference with regards to CD276 in H-scores between HER2^+^ and HER2^−^ cases (Fig. 1F). Possible factors contributing to the differences between HER2 and CD276 mRNA versus protein levels may include post-transcriptional regulation of CD276 protein expression [37].

**Table 1:** Expression of CD276 in breast cancer analyzed by IHC

Stained (n)	Positive (%n)	CD276 staining intensity			
		3 (%n)	2 (%n)	1 (%n)	0 (%n)
25	100/25	40/10	60/15	0/0	0/0

**Fig. 2:**
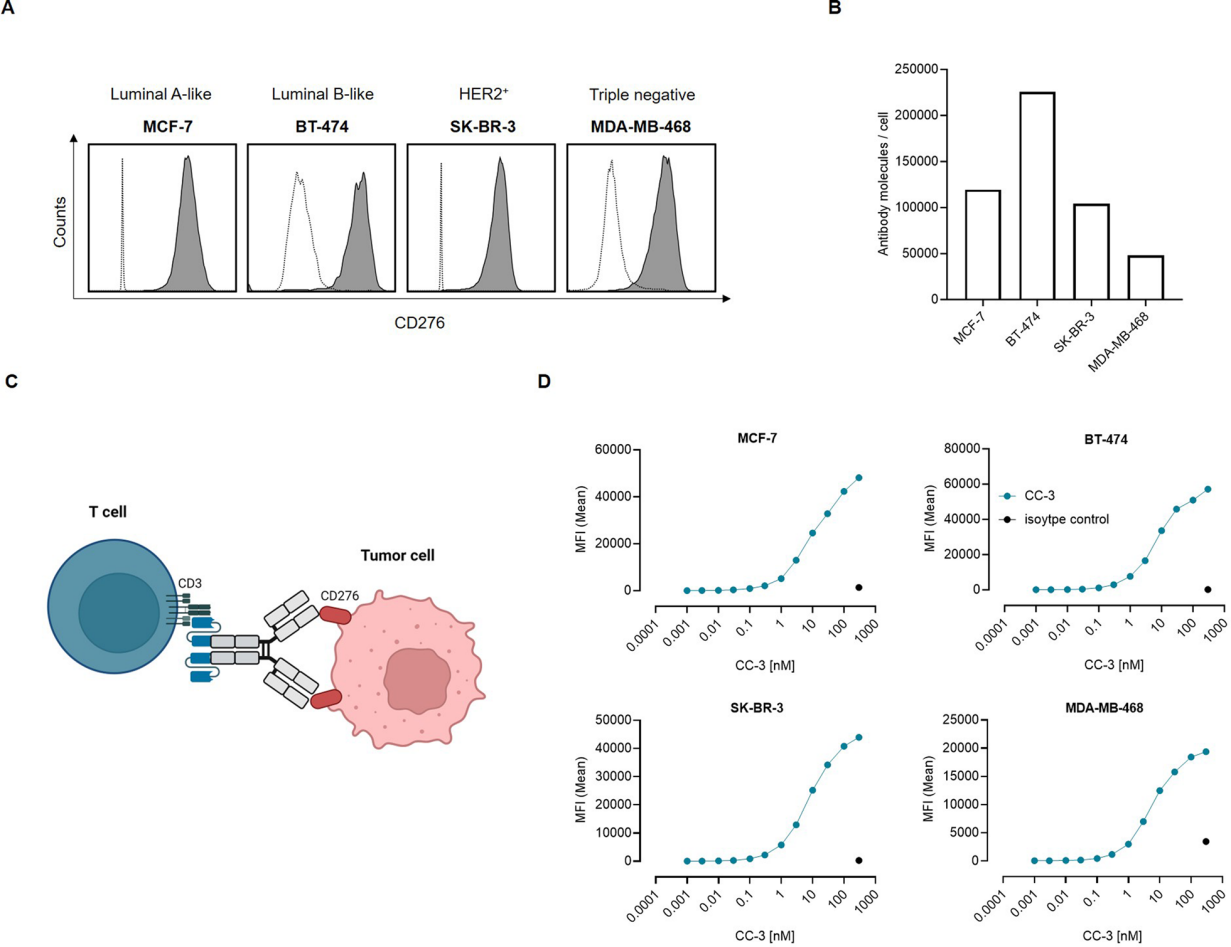
Characterization of CD276 expression and CC-3 as therapeutic compound in BC cells. **A** The indicated cancer cell lines were incubated with a murine CD276 mAb (clone 7C4), then with an anti-mouse PE conjugate followed by flow cytometric analysis. Illustrated is the CD276 expression on the BC cell lines MCF-7, BT-474, SK-BR-3 and MDA-MB-468 (shaded peaks: anti-CD276; open peaks: control). **B** CD276 molecules per cell were quantified in BC cell lines using FACS. Results from two independent experiments are presented. **C** Schematic illustration and mechanism of action of the CD276xCD3 bsAb CC-3. The graphic was created using BioRender (BioRender.com).** D** The indicated tumor cells were incubated with increasing concentrations of CC-3 or the respective isotype control, followed by an anti-human PE conjugate. Binding of the constructs to the indicated cell lines was analyzed by flow cytometry. MFI: mean fluorescence intensities

We then analyzed CD276 surface expression on BC cell lines of four different molecular subtypes: luminal A-like, luminal B-like, HER2^+^, and triple-negative. High surface expression levels were observed with all four cell lines (Fig. 2A). The number of CD276 molecules per cell was between 48,016 and 225,348 (Fig. 2B). Next, we investigated the binding of our CD276xCD3 bsAb CC-3 to the BC cancer cells. CC-3 comprises the variable domains of 7C4 in our previously described IgGsc bsAb format with the single chain sequence of CD3 binding clone M18 as effector part (Fig. 2C) [30]. With all cell lines we observed concentration-dependent binding of CC-3 in flow cytometric titration experiments (Fig. 2D), whereas no unspecific binding to CD276 knockout (KO) cells was observed (Supplemental Figure S2A, B).

### CC-3 modulates T cell synapse formation and activation against tumor cells

**Fig. 3:**
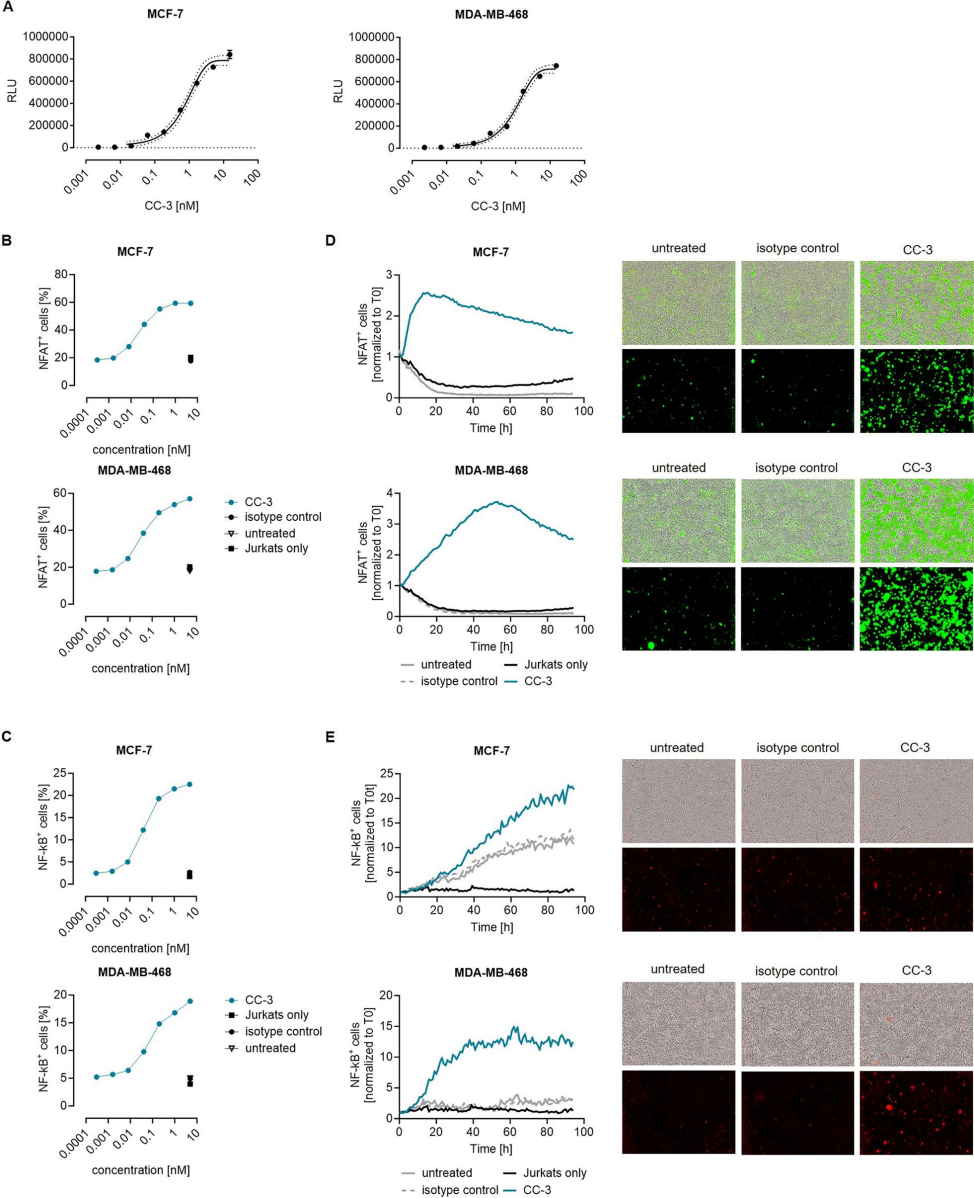
Induction of T reporter cell signaling mediated by CC-3. **A** TCR/CD3 effector cells were cultured with the indicated BC cell lines in the presence or absence of increasing concentrations of CC-3. After 6 h of incubation, luminescence induction was analyzed. The data are presented as mean ± SD. **B**–**E** Jurkat J76TPR reporter cells were cultured with the indicated BC cell lines at an E:T ratio of 2.5:1 with or without CC-3 or the respective isotype control. After 24 h, induction of reporter genes **B** NFAT and **C** NF-κB was analyzed by flow cytometry. **D**, **E** Induction of** D** NFAT and** E** NF-κB was determined through live cell imaging. The left panels show the results for a 94-h time period, while the right panels show exemplary images of the imaging endpoint

Next, we determined the capacity of CC-3 to trigger T cell responses against breast cancer cells. Target cell-bound CC-3 was capable of inducing TCR/CD3 engagement in a concentration-dependent manner, as shown by a T cell activation bioassay (Fig. 3A). Next, we used the Jurkat reporter cell line J76TPR [35] to determine CC-3 mediated induction of the transcription factors NFAT (GFP) and NF-κB (mCherry). To this end, the Jurkat reporter cells were incubated with the indicated BC cell lines with or without CC-3 treatment. Flow cytometric analysis after 24 h revealed an increase in the percentage of NFAT^+^ (Fig. 3B) and NF-κB^+^ (Fig. 3C) Jurkat cells depending on the applied CC-3 concentration. The observed increase of NFAT and NF-κB in Jurkat reporter cells was confirmed by live cell imaging over a period of 94 h (Fig. 3D, E).

**Fig. 4:**
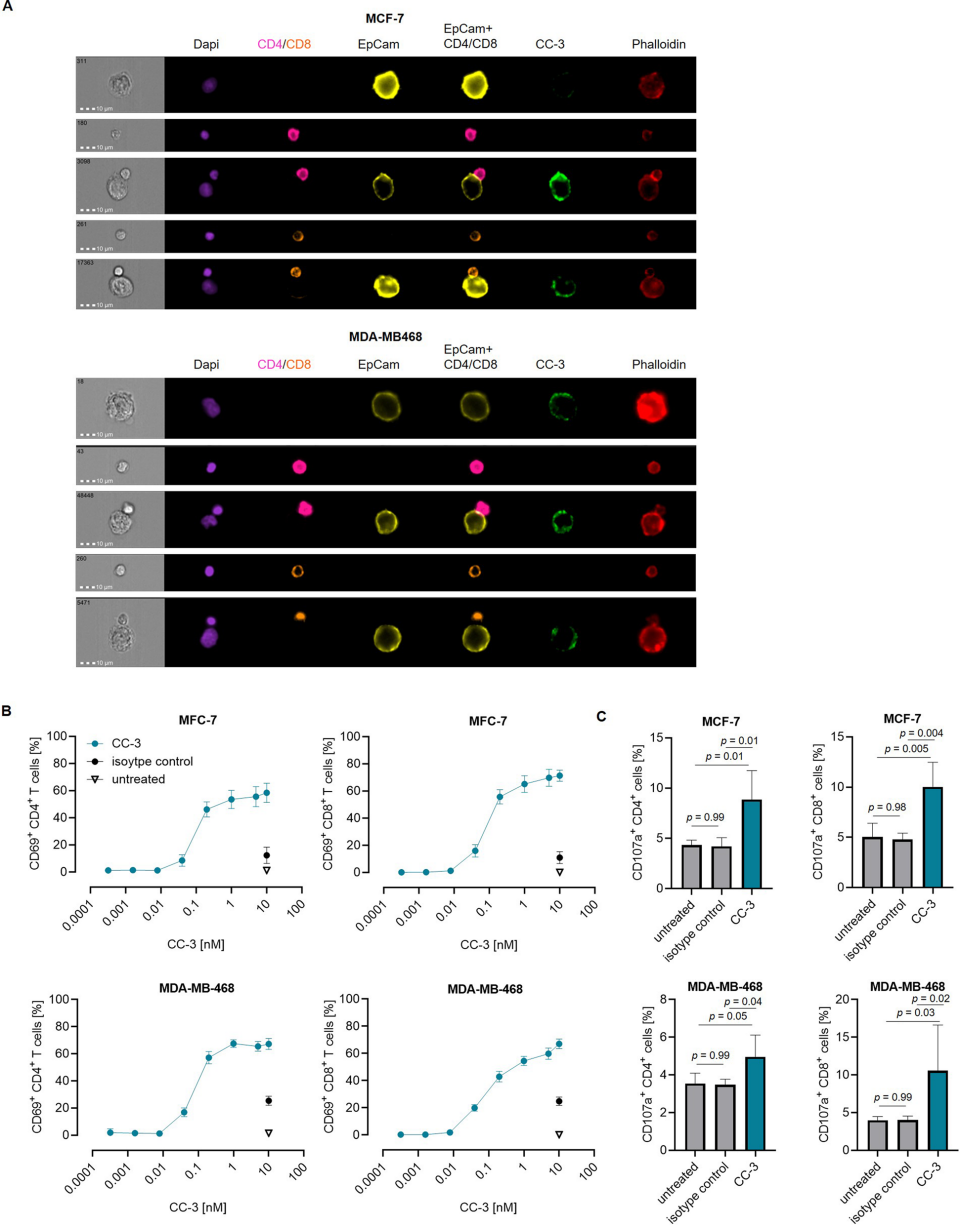
Induction of T cell colocalization and activation against BC cells by CC-3. PBMC were incubated with the indicated tumor cell lines at an E:T ratio of 5:1 in the presence or absence of CC-3 or an isotype control. All constructs were used at a concentration of 1 nM unless otherwise specified. **A** After two hours of incubation, cells were stained with fluorescent-labeled markers and an anti-human Fc-specific antibody. Fluorescent-labeled Phalloidin was added after fixation, and DAPI was used for counterstaining. Analysis was performed using amnis ImageStream mk II. The co-localization of tumor cells and CD4^+^ or CD8^+^ T cells was analyzed by gating on doublets. The formation of the immune synapse between T cell and target cell doublets was analyzed by measuring colocalization in the overlapping region of polarized phalloidin. The scale bar is 10 µm. **B** CD4^+^ and CD8^+^ T cell activation was assessed by flow cytometric analysis of CD69 expression after 72 h. The results represent combined data obtained from PBMC of three independent donors. **C** Degranulation of CD4^+^ and CD8^+^ T cells was determined by analysis of expression of CD107a after 4 h**.** The results represent combined data obtained from PBMC of four independent donors. The data are presented as mean ± SD

We then investigated whether CC-3 promotes colocalization of T cells and tumor cells and the formation of an immune synapse. PBMC were incubated with the indicated BC cell lines and CC-3. Colocalization of tumor and T cells was analyzed by gating on doublets, and binding of CC-3 was visualized by staining with a fluorescence-conjugated anti-human mAb. Immune synapse formation was determined by Phalloidin staining. CC-3 was found to bind tumor cells in all colocalized cases and caused formation of an immune synapse (Fig. 4A, Supplemental Figure S3).

Next, we determined the ability of CC-3 to induce T cell reactivity against BC cell lines. PBMC were cultured with the indicated BC target cells in the presence or absence of increasing concentrations of CC-3. Flow cytometric analysis of CD69 expression revealed maximum activation of CD4^+^ and CD8^+^ T cells by CC-3 concentrations between 1 and 5 nM (Fig. 4B), but not when using CD276 KO cells (Supplemental Figure S2C). Analysis of T cell degranulation as determined by analysis of CD107a expression confirmed that CC-3 strongly stimulated T cells. Statistically significant effects were observed with doses as low as 1 nM (Fig. 4C).

### CC-3 induces T cell proliferation and memory formation

**Fig. 5:**
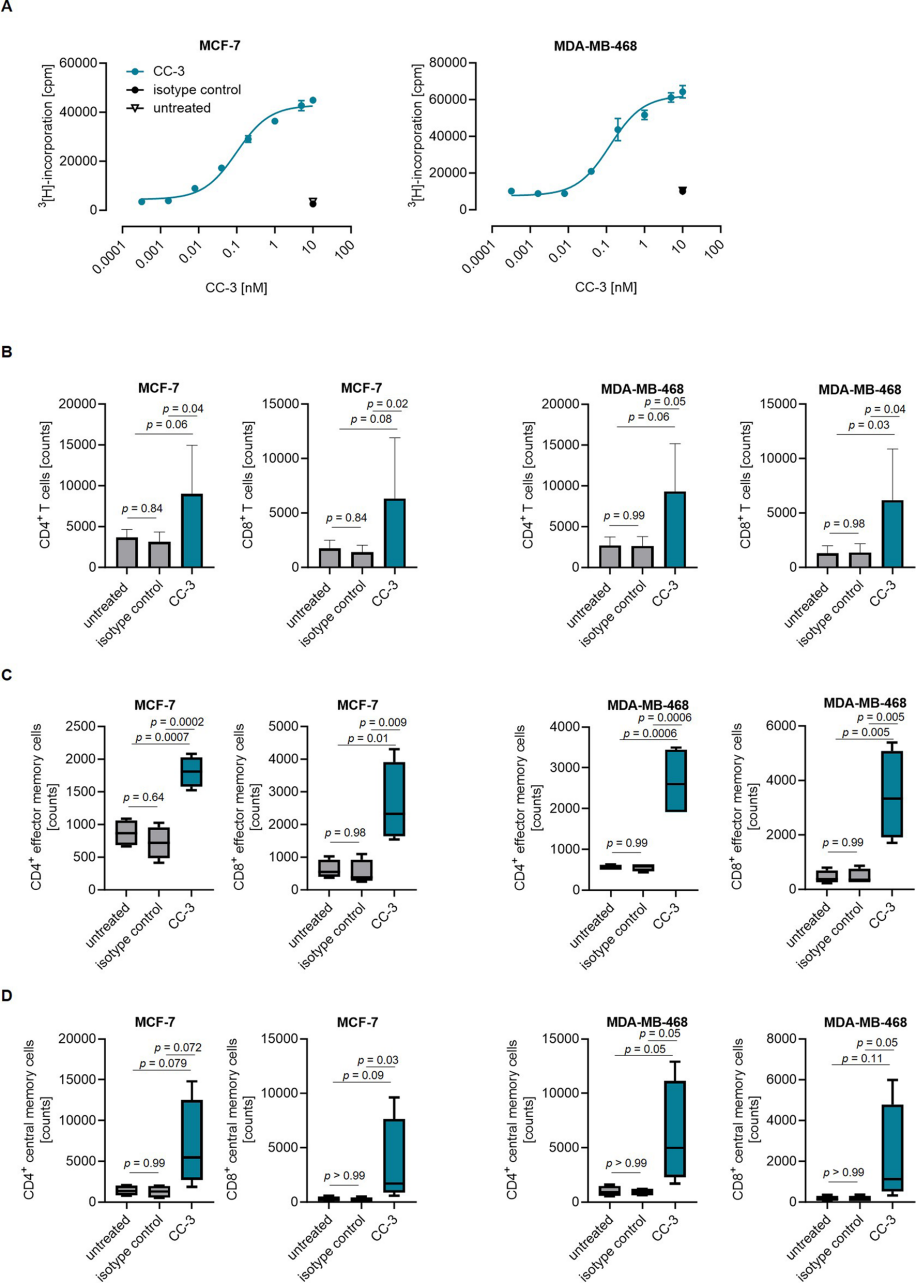
Induction of T cell proliferation and memory T cell populations by CC-3. **A** PBMC were incubated with CC-3 or respective controls at the indicated concentrations in the presence of irradiated BC cells for 72 h (E:T 2:1). The dose response of CC-3 (T cell proliferation) was assessed by thymidine-uptake assays. The results presented are exemplary for one PBMC donor from experiments with PBMC of four independent donors. **B**–**D** PBMC were incubated with CC-3 (1 nM) or isotype control in the presence of MCF-7 or MDA-MB-468 cells (E:T 5:1). After 72 h, fresh target cells and the respective treatment were added to PBMC for an additional 72 h. **B** On day 6, flow cytometric analysis was used to determine the cell counts of CD4^+^ and CD8^+^ T cells. **C**–**D** On day 6, subpopulations of CD4^+^ and CD8^+^ T cells were determined by flow cytometric analysis. CD62L^−^CD45ro^−^ T cells were defined as effector T cells, while CD62L^+^CD45ro^−^ T cells were defined as naive T cells. **C** CD62L^−^CD45ro^+^ T cells were defined as effector memory T cells, and **D** CD62L^+^CD45ro^+^ T cells were defined as central memory T cells. The data are presented as mean ± SD

Given that T cell proliferation is a crucial requirement to combat high tumor burden, we subsequently assessed proliferation following co-culturing T cells with the indicated BC cell lines using a ^3^H-thymidine-incorporation assay after incubation for 72 h. Substantial T cell proliferation was observed in samples treated with CC-3, but not with the isotype control (Fig. 5A). In line, flow cytometric analysis after 6 days of co-culture revealed a significant increase in effector cell numbers for both CD4- and CD8-positive T cells upon incubation with CC-3, but not with the isotype control (Fig. 5B). As memory T cells are the subset most relevant for therapeutic success [38, 39], we investigated which T cell subsets were undergoing proliferation. Flow cytometric analysis showed that CC-3 significantly promoted the expansion of effector memory (Fig. 5C) and central memory T cells (Fig. 5D) with both CD4- and CD8-positive T cells with only marginal effects on naive and effector T cell counts (Supplemental Figure S4A-C).

### CC-3 induces T cell reactivity against breast cancer cells

**Fig. 6:**
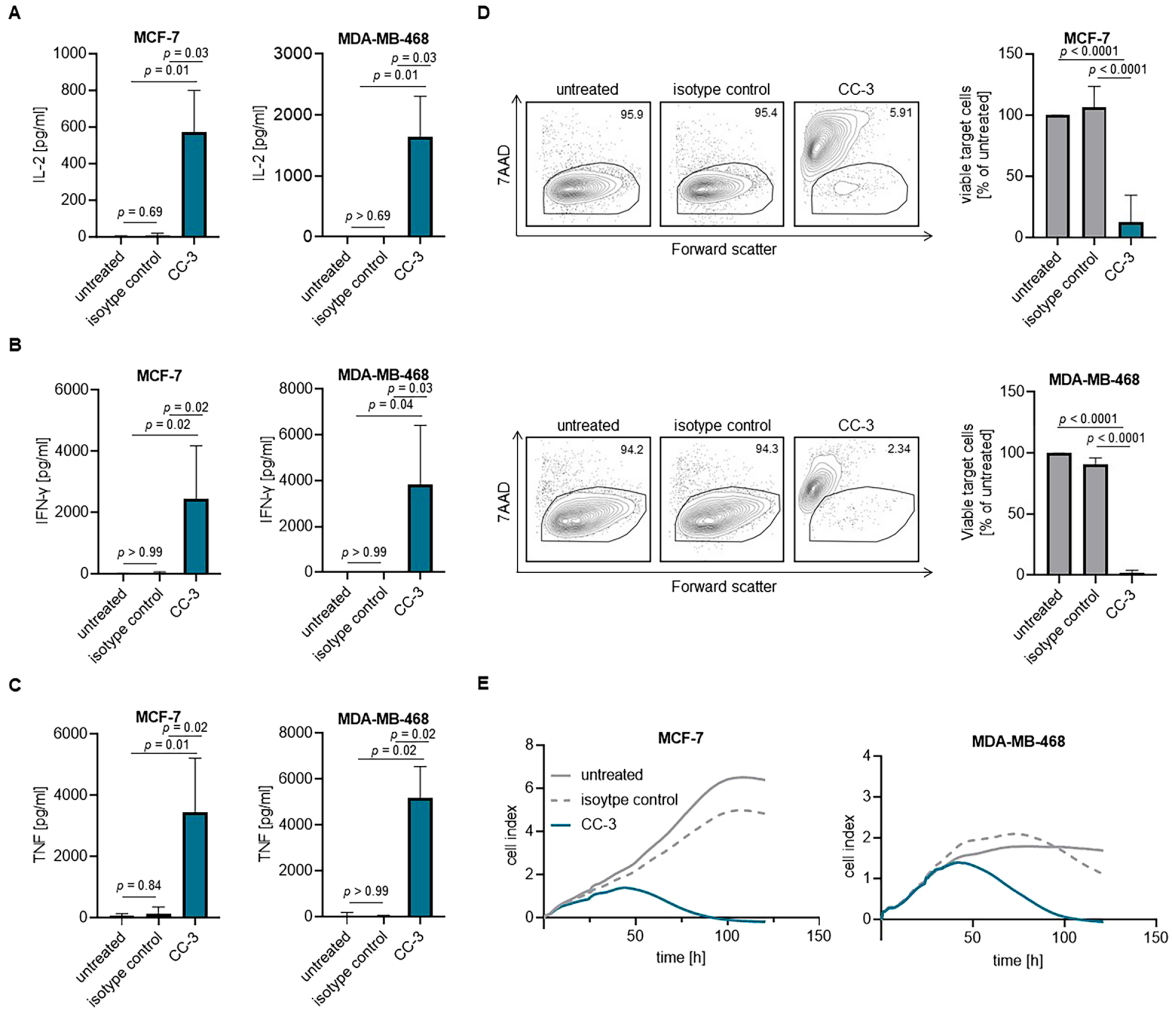
Induction of breast cancer cell lysis mediated by CC-3. PBMC were incubated with the indicated tumor cell lines (E:T 5:1) in the presence or absence of CC-3 or isotype control (1 nM each). The levels of **A** IL-2, **B** IFN-γ and **C** TNF in culture supernatants were measured after 24 h using Legendplex assays. The combined data obtained from PBMC of four independent donors are presented. The data are presented as mean ± SD. **D** Cell lysis of MCF-7 and MDA-MB-468 was determined using a flow cytometry-based assay after 72 h. The left panels show exemplary dot plots, while the right panels display combined data obtained from PBMC of four independent donors. The data are presented as mean ± SD. **E** The long-term cytotoxic effects of PBMC from four independent donors against MCF-7 and MDA-MB-468 cells were determined using the xCELLigence system. The data are presented as mean

As cytokines regulate key aspects of T cell function such as proliferation and effector differentiation, we next analyzed whether T cell activation by CC-3 was mirrored by a corresponding effect on cytokine release. To this end, PBMC were co-cultured with target cells and treated with CC-3 or isotype control (1 nM each). Analysis of culture supernatants after 24 h by Legendplex assays showed a significant increase of the immune effector cytokines IL-2, IFN-γ and TNF (Fig. 6A–C). No effect was observed when using CD276 KO cells, again confirming target cell restricted activity of our bsAb (Supplemental Figure S2D).

To evaluate the capacity of CC-3 to induce tumor cell killing, we analyzed the number of viable tumor cells after 72 h of co-culture with PBMC using flow cytometry-based assays. Our findings revealed that treatment with CC-3 induced a significant reduction in the number of viable tumor cells, whereas no effect was observed with the isotype control, demonstrating that the effect of CC-3 is dependent on target antigen binding (Fig. 6D). Finally, to analyze whether CC-3 exerts cytotoxic effects over extended periods of time which is required for sustained efficacy, long-term killing assays were performed using the xCELLigence system for 120 h. Despite the differences in CD276 surface expression, CC-3 induced strong tumor cell killing in both BC cell lines used (Fig. 6E). Again, analyses with CD276 KO cells confirmed target antigen-specific efficacy of CC-3 (Supplemental Figure S2E).

Hence, CC-3 showed robust anti-tumor efficacy in preclinical settings, suggesting the potential for powerful clinical anti-tumor efficacy in BC.

## Discussion

In recent years, treatment of BC has undergone profound changes, driven amongst others by introduction of immunotherapy, particularly HER2-targeted therapies and ICIs.

The HER2-blocking mAb trastuzumab has become a standard treatment for HER2^+^ BC and has significantly improved outcome for patients [40, 41]. However, a significant number of patients develop therapeutic resistance and disease recurrence [42]. In addition, trastuzumab has shown minimal effects on HER2 medium- and low-expressing cancer cells [43]. As early studies have shown that antibodies targeting multiple domains in HER2 can exert synergistic antitumor effects, a second HER2 mAb, pertuzumab, was developed [44]. As efficacy of trastuzumab relies in part on antibody-dependent cellular cytotoxicity (ADCC), a novel HER2 mAb, margetuximab, that targets the same epitope as trastuzumab but has an optimized Fc domain with in turn improved ability to induce ADCC, was developed. However, Phase III clinical study results did not demonstrate improved overall survival compared to trastuzumab, despite the improved immunostimulatory function [45]. Antibody–drug conjugates (ADCs), which combine a HER2-targeted mAb and a cytotoxic drug, like the FDA-approved compounds trastuzumab emtansine (T-DM1) and fam-trastuzumab deruxtecan (T-DXd) showed significant improvement in progression-free and overall survival compared to standard chemotherapy, thus improving BC patient prognosis [46–49]. Despite these recent advances, HER2-targeted therapies still have a major limitation as they are not suitable for patients with HER2^−^ BC.

Treatment with ICIs has shown promising outcomes in BC patients, particularly in TNBC [11, 12]. However, many patients are not eligible for this T cell-based therapy, and of the eligible ones many do not respond to treatment or for limited time only [13]. Therefore, there is a high clinical need for new therapies, in particular for patients with TNBC, where treatment options remain limited. One potential strategy is bsAb therapy, which may be an effective alternative immunotherapeutic approach to deploy T cells against tumor cells.

CD276/B7-H3 belongs to the B7 family and is considered a promising target for cancer immunotherapy. CD276 is highly expressed on differentiated tumor cells as well as cancer-initiating cells, tumor-associated vasculature, and stroma [15]. Despite the fact that the CD276 receptor is yet not defined, accumulating evidence indicates that CD276 promotes tumor progression. Previous studies have shown that CD276 suppresses essential signaling pathways in T cells, including NF-κB, NFAT, and AP-1, leading to a dampening of T cell activity. In turn, inhibiting CD276 was found to enhance T cell activation in murine models, indicating its potential as a therapeutic option [50]. The frequent expression of CD276 on BC cells makes it a promising target for BC including TNBC [20, 51, 52], where CD276 expression was found to be associated with immune-cold features and collagen accumulation. In turn, it was shown that a mAb targeting CD276 significantly inhibited TNBC growth in mice and reversed the immune-cold phenotype [51].

We have recently reported on the preclinical characterization of CC-3, an optimized CD276xCD3 bsAb for the treatment of gastrointestinal cancers [29, 30] We demonstrated that targeting a membrane-proximal CD276 epitope allowed to use an affinity-attenuated CD3 binder allowing for reduced cytokine induction without reducing desired efficacy. Comprehensive in vitro analyses further demonstrated that CC-3 potently mediates T cell activation, proliferation, and memory formation as well as tumor cell killing. This was confirmed in murine models, where CC-3 did not induce undesired off-target T cell activation and mediated potent antitumor effects with regard to prevention of lung metastasis and flank tumor growth as well as elimination of established tumors using immunocompromised mice adoptively transferred with human PBMC [30].

Upon analyzing tissue sections from 25 BC cases, we here observed membranous staining for CD276 in all cases with no significant difference between HER2^+^ and HER2^−^ cases. Based on these results and the aforementioned data on the pathophysiological role of CD276, we concluded that CC-3 would be a promising compound for BC treatment.

Analysis using J76TPR cells revealed that CC-3 triggers engagement of TCR/CD3 and increases the expression of the downstream signaling targets NFAT and NF-κB. We further found that CC-3 treatment induced formation of tumor-T cell synapses and pronounced T cell activation and degranulation. CC-3 induced T cell effector functions like cytokine release and led to significant tumor cell lysis in both short- and long-term in vitro settings. In addition, CC-3 was found to promote T cell proliferation and memory formation, which are particularly crucial to combat high tumor burden and achieve thorough therapeutic success [29, 38, 39].

One of the main challenges of antibody-based immunotherapy is that target antigens are expressed in healthy tissues, which can result in side effects. CD276 is highly expressed in tumor tissues with expression in normal tissues being minimal. CD276 is weakly expressed on different immune cell subsets [53]. Low expression of CD276 has further been reported in healthy liver tissue. However, significant anti-tumor activity without associated toxicity has been demonstrated in preclinical studies using therapeutic strategies targeting CD276 [16, 31, 54]. When it comes to clinical application, MGD009, a CD276xCD3 dual affinity body, was investigated in solid tumors and temporarily halted due to hepatic adverse events, but the trial resumed after safety evaluation (NCT02628535). Beyond side effects due to effects on healthy tissues, immune-related side effects, in particular the so-called cytokine release syndrome (CRS) represent a major challenge for immunotherapy in general, and for bsAbs in particular. For the latter, tumor burden and initial treatment dose constitute critical factors (55). In this context, heterogeneous target antigen expression amongst different patients, but also within the same tumor constitute challenges that need to be addressed when it comes to defining safety and efficacy of a given bsAb. Notably, safety/side effects of bsAbs are also largely dependent on the CD3 affinity of a given construct [56–58]. In turn, fine tuning CD3 affinity may allow to decouple efficacy from cytokine release, at least to some extent [59, 60]. CC-3 has, as described above, been developed using a CD3 binder with attenuated affinity, and it can be hoped that this results in fewer/reduced side effects upon clinical application. The FIH trial evaluating CC-3 was initially conducted as an open-label, multi-center Phase I clinical trial in patients with metastatic CRC. The study consists of a dose-escalation part to determine the maximum tolerated dose, followed by a dose-expansion part to determine the recommended Phase II dose and provide initial evidence of efficacy. Based on the data provided in this manuscript, the regulatory authorities very recently granted approval to also include patients with breast cancer recruitment of which is meanwhile ongoing (NCT05999396).

## Conclusion

In summary, our study demonstrates that CD276 is highly expressed on BC tissue, and treatment with the CD276xCD3 bsAb CC-3 effectively induces T cell anti-tumor immunity against BC target cells. Our findings characterize CC-3 as a promising immunotherapeutic agent for BC patients and provided the basis for the evaluation of CC-3 in a phase I clinical study (NCT05999396) that very recently started to recruit patients with breast cancer.

## Supplementary Information

Supplementary Material 1.

## Data Availability

The datasets supporting the conclusions of this article are included within the article.

## Abbreviations

7-AAD: 7-Aminoactinomycin D
ADCC: Antibody-dependent cellular cytotoxicity
BC: Breast cancer
BCMA: B cell maturation antigen
bsAb: Bispecific antibody
BSA: Bovine serum albumin
CAR: Chimeric antigen receptor
CD: Cluster of differentiation
DAPI: 4,6-Diamidin-2-phenylindol
E:T: Effector to target
EU: Endotoxin
FDA: Food and Drug Administration
FFPE: Formalin-fixed paraffin embedded
GEPIA: Gene Expression Profiling Interactive Analysis
H&E: Hematoxylin and eosin
HER2: Human epidermal growth factor receptor 2
HR: Hormone receptor
ICI: Immune checkpoint inhibition
IFN-γ: Interferon-γ
IgG1: Immunglobulin G1
mAb: Monoclonal antibody
MFI: Mean fluorescence intensity
PFA: Paraformaldehyde
PBMC: Peripheral blood mononuclear cells
PE: Phycoerythrin
RT: Room temperature
SD: Standard deviation
TCGA: The Cancer Genome Atlas
TME: Tumor microenvironment
TNBC: Triple-negative breast cancer
