# Supplementary material for: Targeting CD276 for T cell-based immunotherapy of breast cancer
Source: J Transl Med. 2024 Oct 4;22:902. doi: 10.1186/s12967-024-05689-4 (PMC11452943; doi:10.1186/s12967-024-05689-4)
Supplement: Supplementary file 1 — Supplementary Material 1. [file 12967_2024_5689_MOESM1_ESM.docx]

**Supplemental Figures and Tables**

**Supplementary Table 1:** Breast cancer patient characteristics

Abbreviations: TNM = tumor, node, metastasis; y = neoadjuvant therapy; p = pathologic stage; c = clinical stage; L = lymphovascular invasion, L1 = yes, L0 = no; V = cancer cells in blood vessels, V0 = no; P = perineural invasion, Pn0 = none, Pn1 = present; X = not specified; NST = breast cancer of no special type; DCIS = Ductal carcinoma in situ, Histological grade based on Elston Ellis score

**
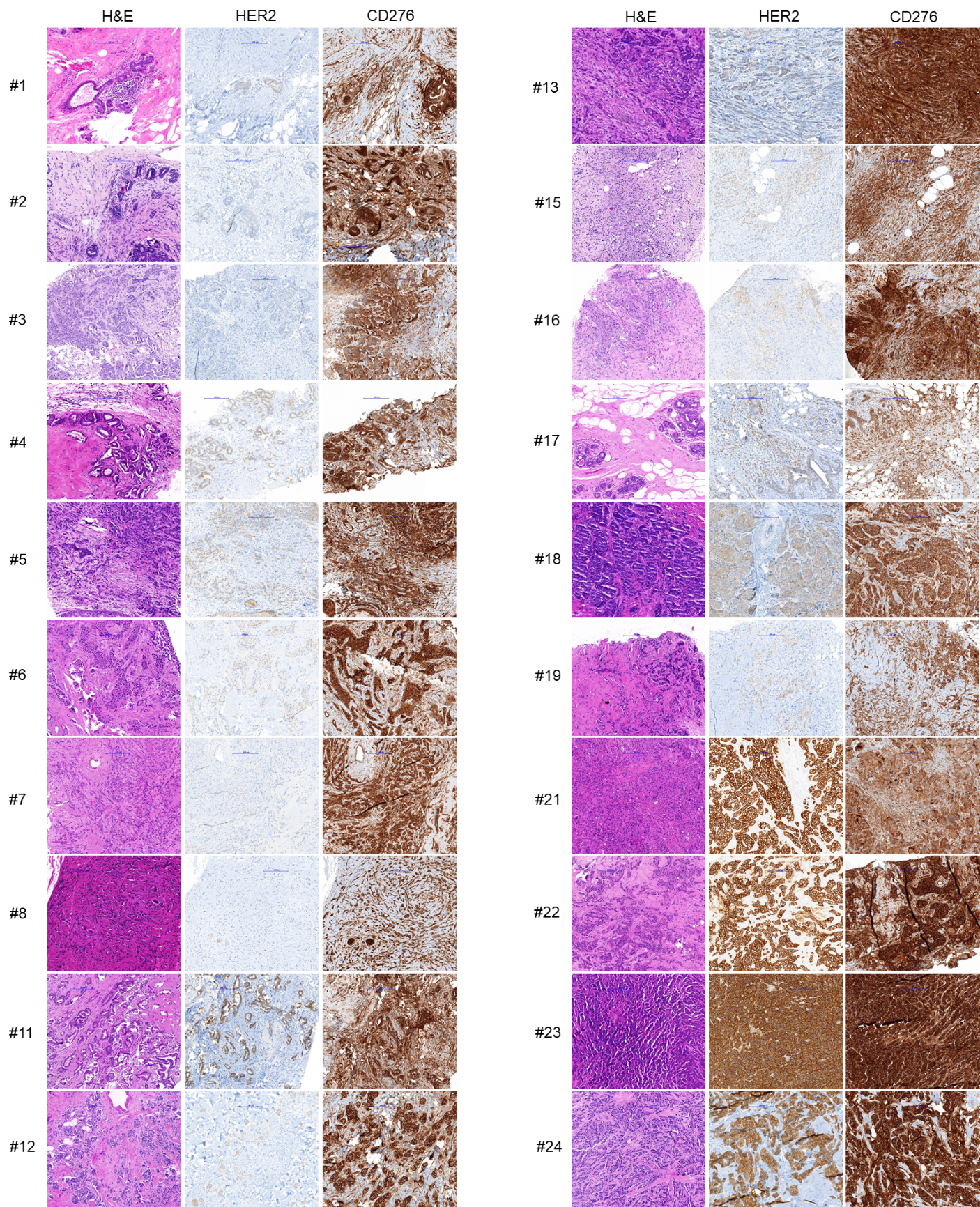
**

**Figure S1: Expression of HER2 and CD276 in breast cancer**

Freshly cut tissues of breast cancer patients were formalin fixed and paraffin embedded. Sections were analyzed for HER2 and CD276 expression by immunohistochemistry. Shown are representative images of tumor tissue sections hematoxylin-eosin (H&E), HER2 and CD276 staining (10x magnification).

**Supplementary Table 2:** Expression of HER2 and CD276 (B7-H3) on n = 25 breast cancer cases by IHC.

IHC-Score for HER2 staining: 0 = negative; 1+ = negative; 2+ = weakly positive (equivocal); 3+ = strongly positive. CD276 staining intensity (IHC-Score): 0 = no expression; 1 = weak, but detectable expression; 2 = intermediate, but clearly positive expression; 3 = strong expression. H-Score: % stained cells x the staining intensity (IHC-Score)

**
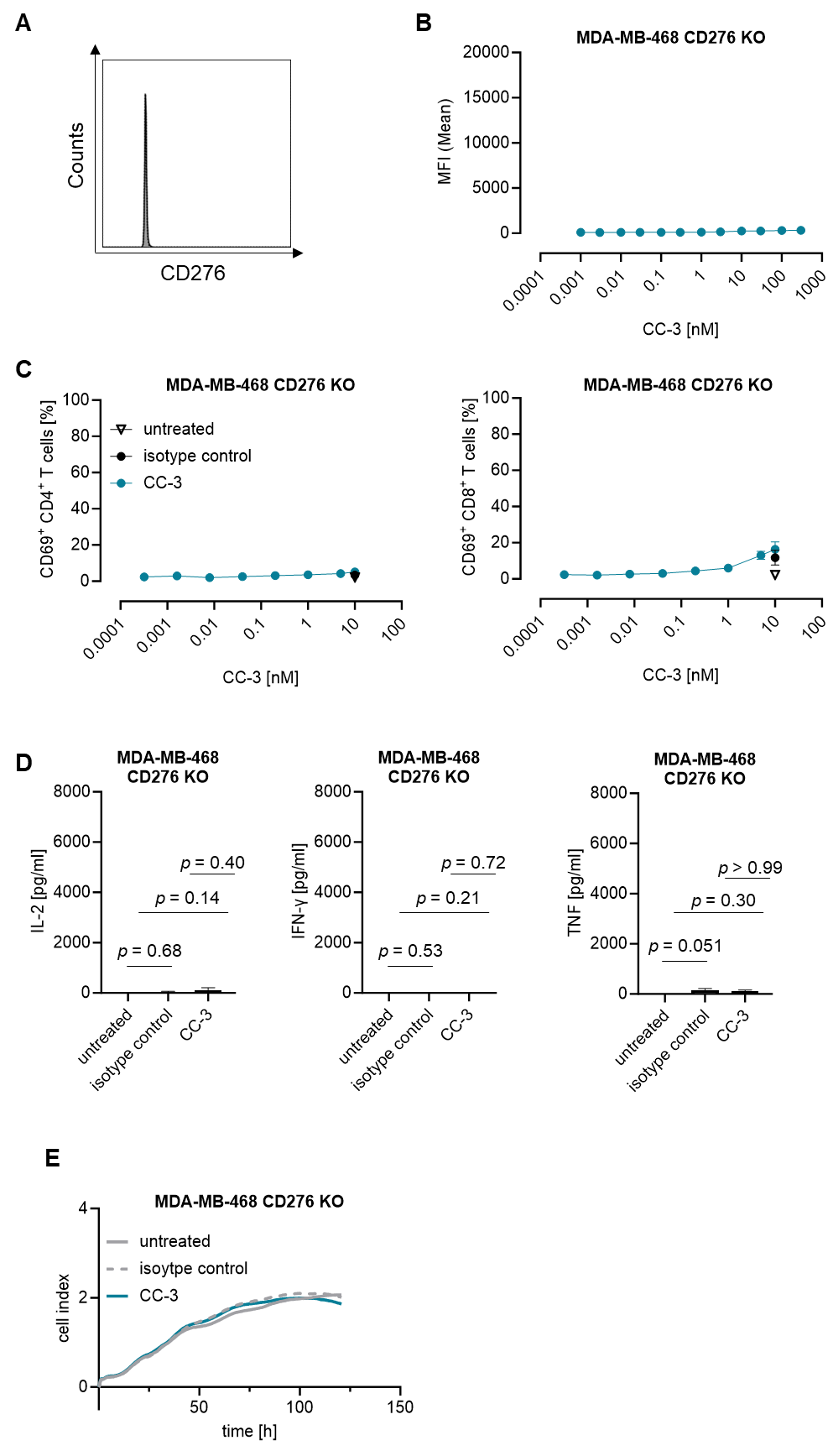
**

**Figure S2: Induction of off-target effects against CD276 knockout breast cancer cells**

**A** MDA-MB-468 CD276 knockout (KO) cells were incubated with a murine CD276 mAb (clone 7C4), then with an anti-mouse PE conjugate followed by flow cytometric analysis (shaded peaks: anti-CD276; open peaks: control). **B** MDA-MB-468 CD276 KO cells were incubated with increasing concentrations of CC-3 or the respective isotype control, followed by an anti-human PE conjugate. Binding of the constructs to the cell line was analyzed by flow cytometry. MFI: mean fluorescence intensities. **C-E** PBMC were incubated with the indicated tumor cell line at an E:T ratio of 5:1 in the presence or absence of CC-3 or an isotype control. All constructs were used at a concentration of 1 nM unless otherwise specified. **C** CD4^+^ and CD8^+^ T cell activation was assessed by flow cytometric analysis of CD69 expression after 72 hours. The results represent combined data obtained from PBMC of three independent donors. **D** The levels of IL-2, IFN-γ and TNF in culture supernatants were measured after 24 hours using Legendplex assays. **E** The long-term cytotoxic effects of PBMC from four independent donors were determined using the xCELLigence system. The data are presented as mean.

**
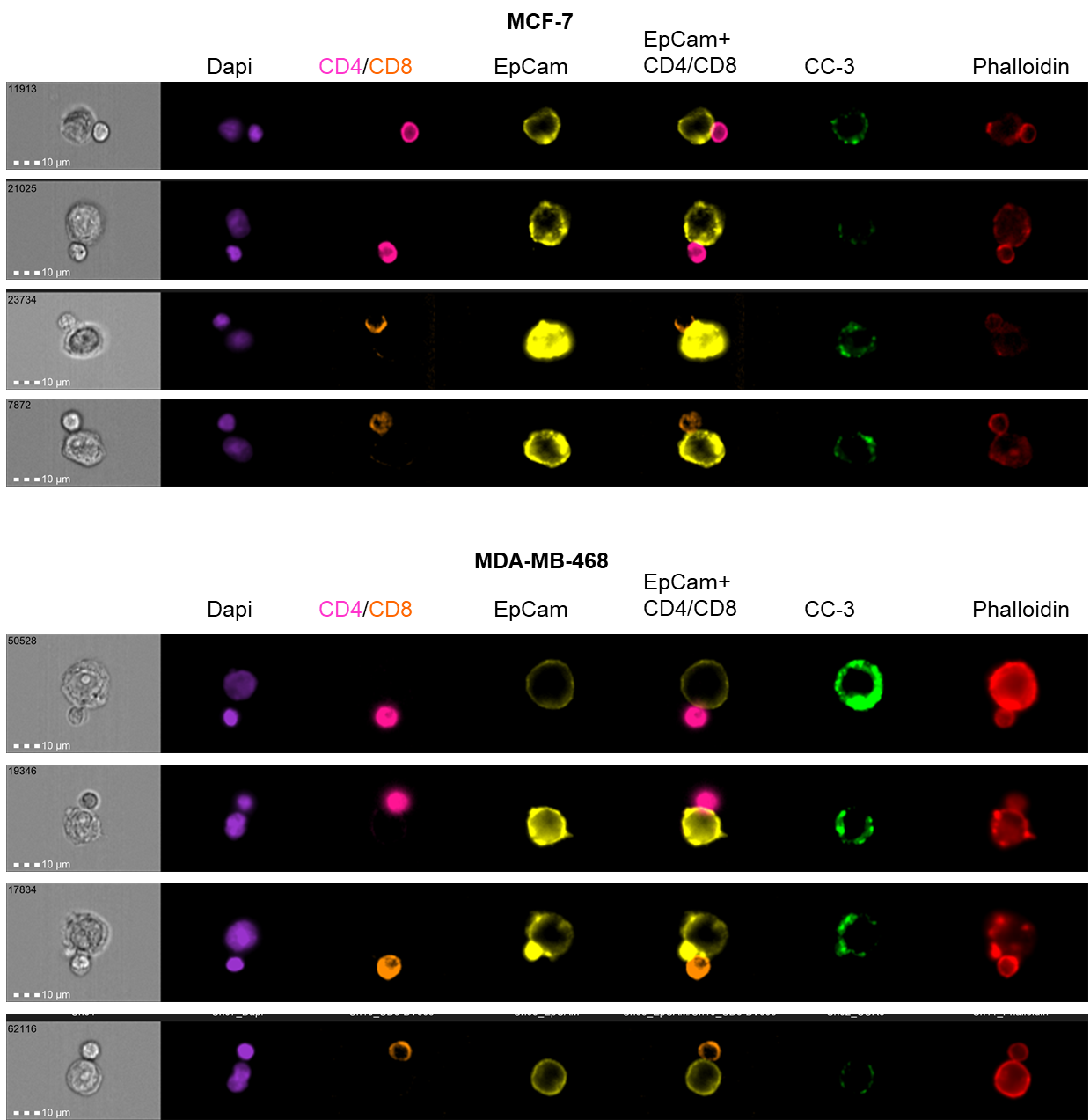
**

**Figure S3: Induction of T cell colocalization and formation of immune synapses**

PBMC from healthy donors were incubated with the indicated tumor cell lines at an E:T ratio of 5:1 in the presence or absence of CC-3 or an isotype control (1 nM each). After a two-hour incubation, cells were stained with fluorescent-labeled markers and an anti-human Fc-specific antibody. Fluorescent-labeled Phalloidin was added after fixation, and DAPI was used for counterstaining. Analysis was performed using an ImageStream. The co-localization of tumor cells and CD4^+^ or CD8^+^ T cells was analyzed by gating on doublets. The formation of the immune synapse between T cell and target cell doublets was analyzed by phalloidin staining. Each marker is depicted at the top of the image panel. Representative images after treatment with CC-3 are shown. The scale bar is 10 µm.

**
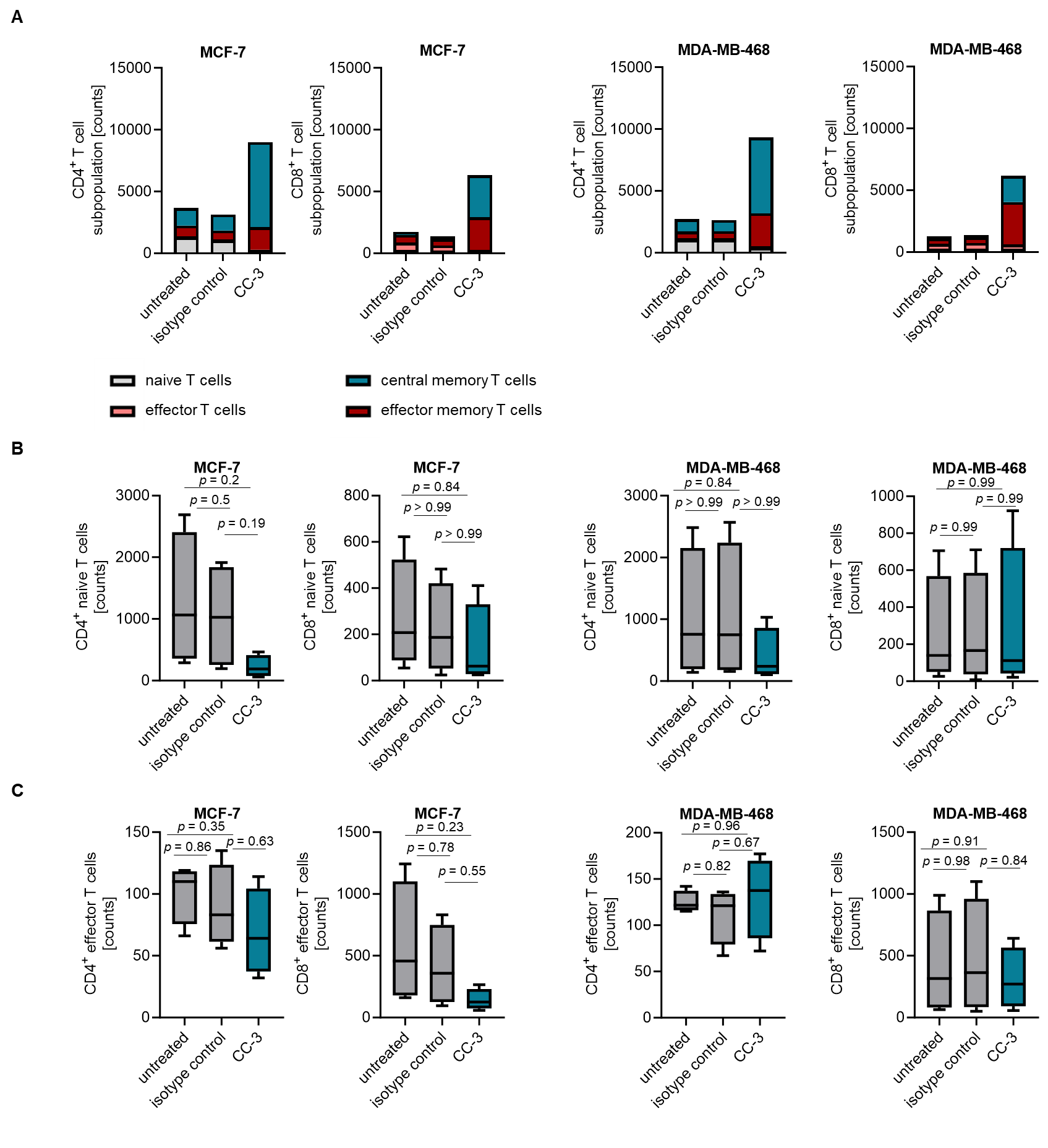
**

**Figure S4: Induction of cytokine secretion and T cell memory formation**

PBMC of healthy donors were incubated in the presence of MCF-7 and MDA-MB-468 cells (E:T 5:1) in the presence or absence of CC-3 or isotype control (1 nM each). **A-C** After 72 h, fresh target cells and the respective treatment were added to PBMC for additional 72 h. On day 6, subpopulations of CD4^+^ and CD8^+^ T cells were determined by flow cytometric analysis. Effector memory T cells were defined as CD62L^-^CD45ro^+^, central memory T cells as CD62L^+^CD45ro^+^, naive T cells as CD62L^+^CD45ro^-^ and effector T cells as CD62L^-^CD45ro^-^. Combined data obtained with PBMC of four independent donors are shown. **A** Pooled data of the distribution of all four T cell subsets. **B-C** Pooled counts of **B** naïve and **C** effector T cells.
